# snoRNAs Offer Novel Insight and Promising Perspectives for Lung Cancer Understanding and Management

**DOI:** 10.3390/cells9030541

**Published:** 2020-02-26

**Authors:** Nour-El-Houda Mourksi, Chloé Morin, Tanguy Fenouil, Jean-Jacques Diaz, Virginie Marcel

**Affiliations:** 1 Univ Lyon, University Lyon 1, INSERM U1052, CNRS UMR5286, Centre Léon Bérard, Cancer Research Center of Lyon, 69373 Lyon CEDEX 08, France; nour.mourksi@gmail.com (N.-E.-H.M.); morinchloe27@gmail.com (C.M.); jeanjacques.diaz@lyon.unicancer.fr (J.-J.D.); 2 Hospices Civils de Lyon Institute of Pathology EST, Site Est-Groupement Hospitalier Est, 69677 Bron, France

**Keywords:** snoRNA, lung cancer, ribosome, rRNA chemical modification

## Abstract

Small nucleolar RNAs (snoRNAs) are non-coding RNAs localized in the nucleolus, where they participate in the cleavage and chemical modification of ribosomal RNAs. Their biogenesis and molecular functions have been extensively studied since their identification in the 1960s. However, their role in cancer has only recently started to emerge. In lung cancer, efforts to profile snoRNA expression have enabled the definition of snoRNA-related signatures, not only in tissues but also in biological fluids, exposing these small RNAs as potential non-invasive biomarkers. Moreover, snoRNAs appear to be essential actors of lung cancer onset and dissemination. They affect diverse cellular functions, from regulation of the cell proliferation/death balance to promotion of cancer cell plasticity. snoRNAs display both oncogenic and tumor suppressive activities that are pivotal in lung cancer tumorigenesis and progression. Altogether, we review how further insight into snoRNAs may improve our understanding of basic lung cancer biology and the development of innovative diagnostic tools and therapies.

## 1. Introduction

Genome-wide analyses unveiled that approximately 80% of the human genome encodes RNAs, while only 1.5% of those are transcribed into proteins. Hence, a majority of RNAs in the cell correspond to non-coding RNAs (ncRNAs), and most of these, including transfer RNA (tRNA), ribosomal RNA (rRNA), microRNA (miRNA), long non-coding RNA (lncRNA), piwiRNA (piRNA) or small nucleolar RNA (snoRNA), are involved in translational regulation [1]. miRNAs are the most studied ncRNAs, and their role in cancer has been extensively studied within the last decades [2]. In contrast, although understanding the biogenesis of snoRNAs and their related molecular mechanisms are still dynamic fields of research 60 years after their first description, the studies dedicated to snoRNAs and cancer have only start to emerge. A computational wide-scale study recently performed in 14 types of cancers on 465 detectable snoRNAs, revealed that human cancer tissues tend to aberrantly overexpress snoRNAs compared to normal paired tissues [3]. Although the roles of snoRNAs have been explored in various cancer types, their exact functions remain incompletely described, particularly in lung cancer.

Lung cancer is the leading cause of cancer-related deaths in women and men worldwide. Tobacco smoking is one of the main risk factors triggering lung tumorigenesis by inducing genomic alterations. Lung cancer diagnosis is an important issue in patient management due to its asymptomatic progression during the early stages. To date, clinical care not only lacks non-invasive tools in order to efficiently and rapidly detect lung cancer onset before the appearance of aggressive symptoms, but does not provide a radical therapeutic arsenal to achieve complete patient remission [4]. Targeted therapies are often confronted with resistance development [5]. Given this observation, lung cancer patient management remains a major public health issue in many countries.

Regarding these medical issues, ncRNAs and especially snoRNAs may provide innovative tools in this field of research. Indeed, though their initial biochemical characterization in the 1990s [6], their association with cancer onset and progression since early 2010s has raised considerable interest. snoRNAs embody on the one hand an innovative biological vision of the disease and on the other, provide original medical management options. In lung cancer, snoRNA investigations are certainly in their infancy and yet they forebode promising clinical opportunities.

This review aims at providing rationale for the pursuit of snoRNA-centered studies in lung cancer. The first sections will present a brief overview of snoRNA biology and lung cancer. The last sections will highlight the clinical relevance of the use of snoRNAs in lung cancer and address their known biological contributions in the molecular mechanisms underlying lung tumorigenesis.

## 2. snoRNAs: An Age-Old Class of Non-Coding RNAs Recently Rediscovered

First described in pea embryos in the late 1950s [7], snoRNAs have been the topic of more than 3200 publications, a third of which were published in the last 4 years due to their central role in the emerging field of epitranscriptomics (PubMed timeline tool). Since then, numerous studies have deciphered in great details the molecular biology of snoRNAs, summarized in the following section. Detailed mechanisms related to snoRNA biogenesis and processing have recently been reviewed by Kufel and Grzechnik [8].

### 2.1. Abundance and Diversity of Human snoRNAs

snoRNAs belong to the ncRNA family and are involved in different cellular processes taking place predominantly within the nucleolus, especially in the processing of rRNAs, messengers RNAs (mRNAs) and other ncRNAs [6,9]. snoRNAs are 60–300 nucleotide long RNAs with complex primary and secondary structures. In 2016, the number of snoRNAs was evaluated at around 600 in humans using both predictions from external databases and de novo predictions [10]. So far, two genomic organizations of snoRNAs have been described, their complexity being correlated with evolution [8,11]. Indeed, this dichotomy in snoRNA biogenesis seems to be correlated with their functions. Around 10% of snoRNAs are transcribed from intergenic regions through dedicated promoters, either as independent or polycistronic genes. These snoRNAs guide cleavage of rRNAs (i.e., SNORA73, SNORD3A (or U3), SNORD14, SNORD22 and SNORD118 (or U8)). This small number of snoRNAs is however vital for cell viability, the lack of rRNA cleavage leading to cell death. In contrast, the remaining 90% of snoRNAs are mainly embedded in spliced introns of host genes, mainly encoding ribosome biogenesis factors or long non-coding RNAs (lncRNAs), and are involved in posttranscriptional chemical modifications of RNAs [11].

There are three main classes of snoRNAs: the small Cajal body-specific RNAs (scaRNAs), the H/ACA box snoRNAs (SNORA) and the C/D box snoRNAs (SNORD). In this review, we will focus on SNORA and SNORD due to their abundance in cells, with around 180 SNORAs and 395 SNORDs predicted in humans [10]. These snoRNAs are composed of conserved box regions to which proteins bind and of a unique 10–20 nt sequence, which provides a high specificity for each snoRNA. The SNORA family is characterized by the H box (ANANNA) and a trinucleotide ACA box, which induce a “hairpin-hinge-hairpin-tail” secondary structure [12]. In vertebrates, SNORAs are able to interact with four proteins, H/ACA ribonucleoprotein complex subunits 1–3 (NHP2, NOP10 and GAR1) and the dyskerin pseudouridine synthase 1 (DKC1) [13,14] to form a small nucleolar ribonucleoprotein (snoRNP). The SNORD family contains a kink-turn (k-turn) structure and conserved sequence boxes C (RUGAUGA) and D (CUGA) at the 5′ and 3′ RNA ends, respectively. In vertebrates, this k-turn structure allows the assembly of the snoRNPs containing nucleolar protein 56 (NOP56), nucleolar protein 58 (NOP58), NHP2-like protein 1 (NHP2L1 or 15.5 Kd) and the rRNA 2′-O-methyltransferase fibrillarin (FBL) [13,15]. Most of the SNORDs have C′ and D′ boxes that provide them with a symmetrical structure, their sequences being similar to C and D boxes [13]. The structure of snoRNPs is essential for the maturation and processing of snoRNAs in the Cajal bodies but also for their nucleolar localization [13]. Indeed, ribonucleoprotein recruitment is crucial for snoRNA stability due to their scaffold activity [16]. It has to be noted that several nomenclatures are currently used to name the snoRNAs. For more clarity, we will use the HUGO Gene Name Nomenclature (HGNC, https://www.genenames.org) that takes in account the molecular family H/ACA box (SNORA) or C/D box (SNORD) in snoRNA naming (Supplementary Table S1). The recent RNAcentral database gives the correspondence between the different nomenclatures (https://rnacentral.org).

### 2.2. snoRNAs Are More Than Just Major Actors of Ribosome Biogenesis

snoRNAs accumulate in the nucleoli where they are responsible for the maturation and posttranscriptional chemical modifications of rRNAs. The ribosome is composed of four rRNAs, three of which are generated from a polycistronic RNA, a 47S pre-rRNA synthesized by the RNA polymerase I (RNA pol I) that contains the sequences of the 5.8S, 18S and 28S mature rRNAs. The fourth rRNA corresponds to the 5S the transcription of which is driven by the RNA polymerase III (RNA pol III). In the 47S pre-rRNA, the three rRNA sequences are separated by external and internal transcribed spacer sequences (ETS and ITS respectively), which are recognized and cleaved by endo- and exonucleases [17]. SnoRNAs were originally described for their role in the endonucleolytic cleavage of rRNAs, demonstrating their essential role in pre-rRNA cleavage [18]. For instance, some SNORDs, such as SNORD3A (U3), are specialized in the cleavage at sites 01 and A0 in ETS in yeast and vertebrate [9,19].

In parallel to their synthesis and cleavage, the three RNA pol I-derived rRNAs that compose the ribosome (28S, 18S and 5.8S) are post-transcriptionally modified. Altogether, they display over 200 chemical modifications that contribute to optimizing activities of the ribosome: 12 base modifications, 95 pseudo-uridylations and 106 2′-*O*-ribose methylations [9,15]. The exact position of the two latter chemical modifications is determined thanks to the unique sequence of snoRNAs. snoRNAs hybridize to rRNAs in a sequence-specific manner and act as guides for enzymes, the pseudouridine synthase DKC1 or the 2′-*O*-methyltransferase FBL, to modify specific rRNA residues. Thus, SNORAs and SNORDs are involved in rRNA pseudo-uridylation [13] and 2′-*O*-ribose methylation [9], respectively.

Recent studies highlighted new roles for snoRNAs in humans. First, several reports indicate that snoRNAs could themselves be cleaved into smaller functional units such as piRNAs [20] and miRNAs [21], thus acquiring novel molecular functions. Second, additional emerging roles are increasingly attributed to snoRNAs, including regulation of mRNA abundance [22] or alternative splicing [23]. Interestingly, these additional functions might reconcile two important observations. On the one hand, it appears that the number of snoRNAs is much greater than that of chemical modifications reported in rRNAs, and on the other hand, some snoRNAs do not present a specific sequence complementary to any known rRNAs making them “orphan” snoRNAs, with unknown functions in ribosome biogenesis. These observations support the existence of yet undiscovered molecular functions for snoRNAs. Thus, the myriad of snoRNAs may display diverse and unexpected functions in cells, in addition to the processing and modification of rRNAs.

### 2.3. Clinical Significance of snoRNAs in Human Diseases

snoRNAs are involved in various pathological processes. Historically, the first snoRNA-associated human disease described is the Prader–Willi syndrome (PWS), a rare genetic disease characterized by hypotonia and hyperphagia [24]. PWS patients lack the locus 15q11.2–q13.1 containing the SNRNP Upstream Reading Frame-Small Nuclear Ribonucleoprotein Polypeptide N (*SNURF-SNRPN*) gene, which hosts six SNORDs (SNORD107, SNORD64, SNORD108, SNORD109a, SNORD115 and SNORD116), the deleterious effects being driven by the absence of SNORDs. Additional examples can be given including the role of snoRNA dysregulation in lipotoxic stress or neurodegenerative disease [25]. Hence, alterations of snoRNAs have a broad impact on human diseases reflecting the required roles of snoRNAs in diverse cellular processes.

Alterations in the expression level of snoRNAs have also been associated with cancer. Dysregulation of snoRNA expression has been reported in different types of cancers, from leukemia [26] to carcinoma [27] and even sarcoma [28]. Due to these alterations in snoRNA expression, these small RNAs have been considered as potential biomarkers in cancer for several years. Indeed, a set of eight snoRNAs can be used to predict the survival rate of gastric cancer patients [29]. snoRNAs share biological features with the extensively studied miRNAs, including the fact they are reliable and detectable in biological fluids, and provide accessible markers useful in the clinic [30]. From the mechanistic point of view, it has to be underlined that alteration of a single snoRNA could have deleterious effects. Several reviews detail the role of snoRNAs in diverse types of cancers, including the ones of Mannoor et al.’s or Thorenoor and Slaby’s [31,32]. We will focus on the role of snoRNAs in lung tumorigenesis. 

## 3. Lung Cancer: A Long Quest to Overcome a Major Killer

### 3.1. A Clinical Overview of Lung Cancer

Being the second most diagnosed cancer worldwide with about 2.1 million new cases in 2018 (GLOBOCAN 2018, WHO, https://gco.iarc.fr), lung cancer remains a major and global public health issue. Around 90%/80% of lung cancers in men/women are caused by tobacco, while the remaining 10%/20% are presumed to be caused by occupational exposure to tumorigenic molecules or spontaneous genetic mutations [33]. Moreover, lung cancer is a highly aggressive disease with a poor 5-year survival rate of about 19%, representing about 1.8 million cancer-associated deaths in 2018 (ACS 2019; GLOBOCAN 2018, WHO).

This poor survival rate is, at least in part, due to the complexity of lung cancer diagnosis at early stages. Indeed, most of the patients are diagnosed at an advanced stage of the disease rendering treatment options limited [4]. The lung contains fewer sensitive neurons compared to other organs. Thus, the lesions have to be severe for the patient to display symptoms, including breathing difficulties. When lung cancer is suspected, imaging techniques are performed after clinical examination. Radiography of the chest is a simple method to detect thoracic lesions. This examination is always followed by the computerized-tomography scan (CT scan) in order to precisely delineate the suspected mass. Then, bronchoscopy is performed to precise the mass localization and if possible, to harvest cytological and histological samples (i.e., bronchoalveolar washing, bronchial brushing and transbronchial biopsy) in order to confirm the presence of neoplastic cells. In case of both peripheral location of the mass and lack of histological malignancy clues using previous methodologies, a nonsurgical transparietal tissue biopsy can be performed using CT-guided fine needle aspiration (FNA) [34]. Although histological analysis of tissue biopsy is the gold standard and is required in most countries to initiate treatment, tissue biopsy could be unfortunately uninformative for few lung cancer patients. Some studies thus investigated the use of liquid biopsy as source of non-invasive diagnostic measures, although they are more suitable as prognostic or predictive biomarkers. For example it has been reported that tumoral cells could be detected using the sputum [35]. Alternatively, blood-derived liquid biopsy containing circulating cancer cells and released DNA–RNA constitutes a wealthy and accessible source of information on tumors. However, clinical relevance of this modern tool has to be demonstrated [36]. 

### 3.2. Histological and Genomic Classification of Lung Cancer

Although deaths associated with lung cancer have been described since the late 19th century, current rates of morbidity and mortality worldwide denote that its management remains difficult for physicians underlining a high tumoral complexity. Lung cancers are classified depending on their histological characteristics. Non-small cell lung cancers (NSCLCs) account for approximately 85% of lung cancers, while the remaining 13% correspond to small-cell lung cancer (SCLC). Among the major groups of NSCLCs, squamous lung carcinomas (lung squamous carcinoma, LUSC, 20% of NSCLCs) are more aggressive than non-squamous lung cancers, which display different molecular backgrounds such as the heterogeneous adenocarcinoma histological subtype (lung adenocarcinoma, LUAD, 63% of NSCLCs) [37]. The histological type is defined based on cell morphology, however when there is a lack of differentiation evidences, immunohistochemistry is performed using adenoma or squamous carcinoma markers: trefoil factor-1 (TTF-1), cytokeratin-7 (CK-7) and napsin-A (ASP4; LUAD); interleukin-9 (IL-9 or p40) and cytokeratin-5/6 (CK-5/6; LUSC) [38,39].

The genomic classification further refines this histological classification at the molecular level, as genomic imbalances are recurrent in lung cancer. Chromosomes impacted by this genomic instability harbor either loss or gain of genes identified as oncogenes in lung cancer [40]. Genome-wide approaches have thus enabled the separation of NSCLCs into different molecular subtypes, based on genomic alterations of driver genes. Genomic alterations mainly correspond to activated mutations on genes encoding kinases, triggering the constitutive activation of signaling pathways to support tumorigenesis and aberrant cancer cell proliferation. Intrinsic mutations on the Kristen Rat Sarcoma Viral Oncogene homolog (*KRAS*) are important alteration that occurs in 25% of lung adenocarcinoma. On other hand, alteration on the Epidermal Growth Factor Receptor (*EGFR*) gene is found in 15% of lung adenocarcinoma and translocations of the Anaplastic Lymphoma Kinase (*ALK*) gene occur in 3%-7% of lung adenocarcinoma [41]. Other genomic alterations (translocation: ROS proto-oncogene 1 (*ROS1*) and Rearranged during Transfection (*RET*); mutation: B-RAF proto-oncogene (*BRAF*), Mesenchymal Epithelial Transition (*MET*) and Erb-b2 receptor tyrosine kinase 2 (*ERBB2*); amplification: *MET*, etc.) also drive lung tumorigenesis but are less frequent [42]. Of note, 40% of lung adenocarcinoma have not yet been associated with particular driver genomic alteration [41]. In this context, the tumor initiation process is unclear, although it likely results from alterations in gene expression.

### 3.3. Therapies and Limitations

Appropriate therapies are chosen depending on the histological and genomic classification of lung tumors and on the stage of the disease. Like most cancers, early treatment considerably improves the survival rate, surgery remaining the main therapeutic option. The optimal treatment of stages I and II lung cancer is tumor resection. When surgery is impractical, patients are recommended to be treated via radiotherapy and/or chemotherapy [35]. However, chemotherapy results in non-specific tissue targeting, attacking both tumor and healthy tissues. Moreover, chemotherapy-induced side effects are often observed and, for some patients, associated with only a moderate tumor response.

In addition to these therapies, due to the great heterogeneity in lung cancer, personalized medicine is a powerful strategy to limit toxicity and improve patient outcome [43]. The classification based on specific driver mutation subtypes enables clinicians to precisely treat tumor cells through targeted therapies. Targeted therapies are small molecules successfully integrated into lung cancer patient management these past decades that mainly target the mutant tyrosine kinases, and are so called tyrosine kinase inhibitors (TKIs). They are used in first and/or second line treatment in patients displaying advanced tumors at diagnosis. For example, *EGFR* mutant-driven tumors that represent 15% of NSCLCs, can be treated by therapeutic molecules designed to specifically inhibit mutant and overactivated EGFR proteins, such as erlotinib, afatinib and gefitinib [41]. Other FDA-approved drugs are routinely used such as crizotinib, ceritinib or alectinib for *ALK* rearranged NSCLC or crizotinib for *ROS1* rearranged NSCLC. Additional drugs are currently under evaluation (MET: crizotinib; RET: cabozantinib; NTRK: entrectinib) [41,44]. However, targeted therapies display some limitations. First, there is no available drug to specifically target the *KRAS* mutant driven tumors, which represent the most important proportion of NSCLCs, and downstream targets are under evaluation for these particular tumors (selumetinib a MEK1/2 inhibitor). Second, although TKIs have a great efficiency compare to non-targeted therapies, most lung cancer patients undergo progression mainly due to resistance. Several mechanisms have been described to explain TKI resistance, including accumulation of genetic alterations within or outside the targeted tyrosine kinase [41]. Such acquired mutations are thought to arise from a latent reservoir of stem-cell like cells tolerant to the drug [45]. For instance, in 50%–60% of cases, treating an *EGFR*-mutant primary tumor with targeted therapies induces a positive selection of resistant cancer cells within the tumor due to the acquisition of a secondary mutation, T790M in the EGFR protein that occurs outside of the ATP binding site [41]. The new generation of TKIs, such as osimertinib, is able to inhibit those resistant *EGFR*-mutant cells but, once again, new mutated cells can be selected.

When no genomic alterations are detected and when at least 50% of tumor cells express the immunosuppressive programmed cell death 1 ligand 1 (PD-L1) membrane protein, immune-checkpoint inhibitors (ICI) are used [46]. These correspond to monoclonal antibodies (e.g., pembrolizumab, atezolizumab or nivolumab) that could be proposed in first or second line treatment to reinforce the effects of radio-chemotherapy. At present immunotherapy is moderately introduced in lung cancer patient management because of the need for sufficient PD-L1 expressing cells [46]. However, results of the KEYNOTE 042 clinical trial demonstrated that patients with locally advanced/metastatic lung cancer expressing low PD-L1 (1% tumor proportion score) benefit from first line pembrolizumab treatment [47]. Hence, new mechanisms to understand lung tumorigenesis and relapse remain to be identified to develop innovative therapies to improve lung cancer patient management. 

## 4. Altered Expression of snoRNAs in Lung Cancer

Several pioneer studies investigated the landscape of snoRNAs differentially expressed during lung oncogenesis, the objective being to identify putative snoRNAs, which could be used as diagnostic biomarkers. To achieve this, most studies focused on overexpressed snoRNAs in lung cancer patients versus healthy donors, both in tissues and circulating fluids.

### 4.1. snoRNA Profiling in Normal and Tumoral Lung Tissues

To our knowledge, Liao et al. [48], in 2010, paved the way for extended snoRNA profiling in lung cancer. Definition of a signature of snoRNAs, the altered expression of which was associated with early stage NSCLC, was achieved through the comparison of the expression level of 352 human mature snoRNAs using a GeneChipR Array. Authors compared snoRNA expression in 22 NSCLC stage I resected tissues (11 LUSC and 11 LUAD) versus paired non-cancerous specimens resected from the same patient at a distance from the tumor. Six snoRNAs (SNORA80E, SNORA73B, SNORD33, SNORD66, SNORD76 and SNORD78) were significantly overexpressed at least 1.5-fold in samples of lung cancer tissue compared to the ones of healthy donors (Table 1). Furthermore, no difference was observed between the expression of these snoRNAs and the two types of NSCLCs, indicating that there was no histological-specific association in this study, which may be due to the small number of samples. Although results were thoroughly validated by RT-qPCR, one has to keep in mind that the Chip array depends on the identification of a selected set of transcripts preventing the analysis of non-included snoRNAs. As a consequence, a Chip array designed to detect 352 correctly annotated snoRNAs oversees around half of the total 600 predicted snoRNAs [10]. To extend this preliminary lung cancer snoRNA profiling, Gao and colleagues [49] proposed to use RNA-seq. This high throughput technology permitted the examination of 458 mature snoRNAs in 12 pairs of normal and tumor lung tissues classified as stage I NSCLC. 29 snoRNAs, including SNORA71A, were significantly overexpressed (by at least 3-fold) in tumor tissues compared to normal biopsies (Table 1).

Since then, snoRNA screening has been preferentially performed by exploiting publicly available RNA-seq datasets, the most commonly used being the ones hosted by The Cancer Genome Atlas (TCGA) database. Recently, Gong et al. [3] investigated the expression of 465 snoRNAs in pairs of tumoral/normal tissues arising from 31 types of cancers, including two main subtypes of NSCLCs (LUAD *n* = 46 and LUSC *n* = 45; Table 1). In contrast to previous studies, they compared the global alteration of snoRNA expression rather than individual snoRNA variations between normal and tumoral tissues. They found that global change in snoRNAs mainly corresponded to their overexpression in 12 cancers. In LUAD and LUSC, a significant (2.5-fold) increase was observed in tumoral compared to normal tissues, making lung cancer the 4th cancer exhibiting the highest snoRNA overexpression. Meanwhile, analysis of aberrant snoRNAs that distinguished female smokers and non-smokers within normal and cancerous tissues from lung adenocarcinoma, identified 28 snoRNAs the expression of which was significantly altered between normal and tumoral tissues, irrespective of the smoking status, reinforcing the finding that alterations in snoRNA expression occur in lung cancer [50] (Table 1). In parallel, using machine learning algorithms, Pan et al. [51] investigated the expression pattern of more than 1000 snoRNAs in 8 cancers including NSCLC (LUAD *n* = 559 and LUSC *n* = 521). They found a specific signature encompassing only a few snoRNAs compared to other types of cancers both for LUAD (SNORD7, SNORD81 and SNORD99) and LUSC (SNORA31A, SNORA47 and SNORD83B). 

**Figure 1:**
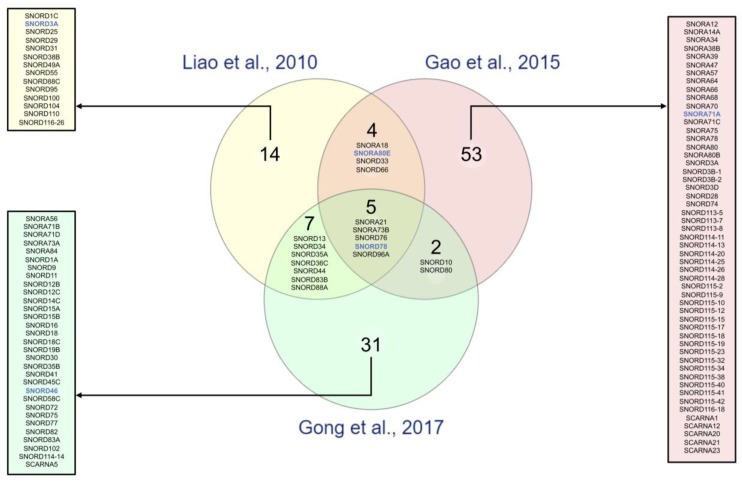
Comparison of snoRNA signatures in primary lung cancer tissues. Three studies identified snoRNAs significantly dysregulated in lung cancer using pairs of cancerous and non-cancerous primary pulmonary tissues at diagnosis using different approaches (yellow, Liao et al., 2010 [48]: microarray; red, Gao et al., 2015 [49]: RNA-seq; and green, Gong et al., 2017 [3]: analysis of public TCGA dataset). The Venn diagram presents the logical relation between the datasets of significantly altered snoRNAs provided by these three studies. Briefly, we uploaded the snoRNA lists provided by each study and identified snoRNAs present either in 1, 2 or 3 datasets using an online visualization tool (http://www.molbiotools.com/listcompare.html). Intersections correspond to snoRNAs commonly dysregulated in at least 2 studies, while boxes present snoRNAs identified in a single study. Interestingly, this comparison reveals a set of 5 commonly detected snoRNAs (SNORA21, SNORA73B, SNORD76, SNORD78 and SNORD96A). SnoRNAs, the roles of which have been investigated in lung tumorigenesis, are indicated in blue.

However, these results display discrepancies in the NSCLC-snoRNA profile obtained despite the high quality of analyses (Figure 1). Such heterogeneity may be due to sampling and patient population diversities, as well as to different approaches employed to track snoRNA footprints in NSCLC pulmonary tissues (Table 1). It may also reflect the heterogeneity of lung cancer itself. Indeed, a lung tumor mass is composed of stromal cells, endothelial cells, cancerous differentiated lung cells that are highly proliferative and another small subpopulation sharing cancer stem cell (CSC) features: the initiating tumoral cells (TICs) also called drug-tolerant persister cells (DTPs) [52]. TICs are capable of self-renewal and appear to be the main source of tumorigenesis onset in vivo [53,54]. Detection of TICs relies on the presence of the CD antigen 133 (CD133) surface marker of cancer stem cells [55], the expression of the cytoplasmic marker (KDM5A or histone deacetylase (HDAC) [56] or the over-activity of the aldehyde dehydrogenase 1 (ALDH1) [57,58]. In 22 NSCLC primary chirurgical pieces, 22 snoRNAs have been identified as mainly up-regulated between ALDH1^+^ and differentiated ALDH1^−^ cells using microarray analysis (Table 1) [59]. SNORA3 and SNORA80E are the top two highly expressed snoRNAs in TICs compared to differentiated lung cancer cells [59].

### 4.2. Circulating snoRNA Profiling in Healthy and Lung Cancer Patients

Extracellular ncRNAs appear to occur through cell death or controlled exocytosis (exosomal RNAs) [61]. Thus, by analogy with prior reports on miRNAs found in blood and sputum in lung cancer [62,63], released circulating snoRNAs were also prospected in biological fluids (blood, plasma, urine and sputum). The presence of snoRNAs within biofluids in cancer was examined for the very first time in NSCLC, lung cancer being a disease for which non-invasive biomarkers are urgently required due to the difficulty in performing tissue biopsies [64]. A proof-of-concept study demonstrated that snoRNAs can be detected in different blood fractions (cells, plasma and pellet/supernatant of plasma) of NSCLC patients using RNA-seq approaches [65].

Liao and al. [48] extended their initial investigation on NSCLC tissues to patient plasma using their primary tumor-related snoRNA signature (SNORA80E, SNORA73B, SNORD33, SNORD66, SNORD76 and SNORD78). Similarly to miRNAs, the panel of these six snoRNAs [48] is resistant to RNAse digestion and stable over time in healthy plasma. snoRNAs could thus be routinely and accurately detected using RT-qPCR. Quantification of circulating snoRNAs demonstrated that only three snoRNAs (SNORD33, SNORD76 and SNORD66) out of the 6-snoRNA signature displayed a significantly higher level in the plasma of cancer patients compared to healthy individuals. In addition, combined detection of these three plasmatic snoRNAs can discriminate NSCLC patients from cancer-free individuals and from chronic obstructive pulmonary disease (COPD) patients with a high sensitivity and specificity, making them strong NSCLC-specific diagnostic candidates. Of note, the analysis of a small cohort (four LUSC samples and three samples from healthy donors) using RNA-seq instead of RT-qPCR, identified seven snoRNAs the level of which was significantly decreased in the plasma of lung cancer patients compared to healthy donors (SNORD2, SNORD38A/B, SNORD58B, SNORD73A, SNORD75 and SNORD81), suggesting that other snoRNAs can improve this plasma-derived snoRNA signature [65].

Moreover using a similar approach, sputum from healthy and NSCLC individuals was also shown to contain stably and reliably measurable snoRNAs [60]. In sputum, exploration of the expression pattern of the 6-snoRNA signature led to the detection of four snoRNAs (SNORA80E, SNORD33, SNORD66 and SNORD78) that significantly discriminated NSCLC patients from cancer-free smokers [60]. As SNORD66 and SNORD78 are commonly overexpressed in lung cancer, examination of their expression level in sputum was carried out and results were combined with examination of miRNA levels that have already been correlated with lung cancer disease. Combination of snoRNA and miRNA detection exhibited stronger sensitivity and specificity than individual ncRNA tests to diagnose lung cancer in sputum [66].

snoRNAs appear to be stable and strong non-invasive biomarkers from a technical point of view. Although these studies focused on the snoRNA signature derived from lung cancer tissues, the correlation of snoRNA levels between tumoral tissues and liquid biopsies derived from the same patients has not yet been investigated. Nevertheless, similarly to tissues, circulating snoRNA levels vary in liquid biopsies of lung cancer patients compared to healthy donors, supporting the alteration of snoRNA expression levels in lung cancer.

### 4.3. Clinical Significance of Altered snoRNAs in Lung Cancer

The profiling of snoRNAs in lung cancer highlighted a set of snoRNAs that where significantly dysregulated, mostly overexpressed, in cancerous versus normal samples, some snoRNAs being specific to cancers compared to other lung diseases or to particular histological subtypes of lung cancer. Hence, detection of these disease-specific snoRNAs could be used as a diagnostic tool for lung cancer. Based on these encouraging findings, studies have focused on the prognostic value of these snoRNA-derived signatures.

Gong et al. [3] based their approach on a wider panel of snoRNAs to evidence their biomedical significance. The 200 snoRNAs displaying the greatest variability in their expression between cancerous and healthy tissues in different cancers were selected. Their expression level was then used to assign each cancer to several subtypes using Consensus Cluster Plus. Finally, survival associated with each subtype was evaluated. Novel subtypes classified according to snoRNA expression showed distinct prognostic value in LUSC, as well as in three other cancers, suggesting that snoRNA profiling might refine histological classification of lung cancers displaying distinct survival rates.

Having identified a list of 29 highly overexpressed snoRNAs in lung tumor tissues, Gao et al. [49] then aimed at highlighting the association between overall survival of lung cancer patients and snoRNA expression. Individual association of each of the 29 snoRNAs analyzed by RNA-seq with survival was first determined using both univariate and multivariate cox regression analyses in a collection of 77 frozen lung cancer tissues corresponding to different grades of the disease. Among these 29 snoRNAs, only three (SNORA47, SNORA68 and SNORA78) were identified as independent markers of poor prognosis after adjustment on clinical standards (age, gender, race, smoking, stage and histology). Patients harboring lung tumors with high levels of each of these three snoRNAs displayed a poorer overall survival than patients carrying tumors with relatively lower levels. Moreover, based on the combined expression level of these three snoRNAs, a validated risk score was extrapolated to predict outcome of NSCLC patients by assigning them to a high-risk or low-risk group. Additional association between individual snoRNA expression and survival was reported. For instance, in 82 NSCLC samples, RT-qPCR evaluation of SNORA3 and SNORA80E expression levels, up-regulated in TICs, revealed that individually their highest expression is statistically associated with poor prognosis. Similarly, high levels of SNORA71A [67] or SNORD50A/B [68] in lung cancer tissues are independent predictors of an overall shorter patient survival.

Although histological analysis of tissue biopsy remains the gold standard of cancer diagnosis, non-invasive prognostic and predictive biomarkers are needed in clinic. The current literature supports the use of snoRNAs as diagnostic biomarkers in plasma and sputum that may offer lung cancer biomarkers’ innovative candidates. Surprisingly, prognostic and predictive significance of the snoRNAs have never been evaluated neither in sputum nor in blood in lung cancer patients.

## 5. snoRNA Dysregulation Contributes to Lung Cancer Tumorigenesis

Several studies have shown differences in snoRNA expression between normal and lung cancer cells in humans, as detailed above. However, few studies have investigated the involvement of snoRNAs in lung tumorigenesis and progression. To date, only seven studies explored the contribution of snoRNAs to lung cancer and mainly by determining their role in cellular processes, the molecular mechanism remaining largely understudied. In this section, we reviewed the list of snoRNAs, which have been shown to be involved in lung tumorigenesis.

### 5.1. SNORA80E Inhibits Apoptosis and Supports Stemness

From the profiling studies, SNORA80E (also termed SNORA42 or ACA42) emerged as a recurrent overexpressed H/ACA box snoRNA in many independent signatures of lung cancer, both in tissue and fluid biopsies (Figure 1) [48,49,59,60]. Moreover, SNORA80E is overexpressed in other cancers, such as colorectal cancer [69]. Chromosomal location of SNORA80E (1q22) in a well-known amplified genomic region in lung cancer [40] argues in favor of a relationship between lung cancer development and SNORA80E overexpression. Two studies from the laboratory of Jiang [59,70] investigated the role of SNORA80E in lung tumorigenesis.

The impact of the modulation of SNORA80E expression on cellular phenotypes in lung cancer cell lines have been elucidated by a gain- and loss-of-function experimental strategy in vitro and in vivo. SNORA80E knockdown using siRNA in H460 and H1944 NSCLC cell lines abolishes cell proliferation 24 h post-transfection onwards and formation of colonies in soft agar by 80%. These severe effects observed in response to SNORA80E knockdown demonstrate its pivotal role in oncogenesis. Consistently, overexpression of SNORA80E via its transfection using a pCMV-SNORA80E vector in normal bronchial epithelial cells (BEAS-2B) and in the H1299 NSCLC cell line promotes cell proliferation and soft agar colony formation. Thus, SNORA80E favors cell proliferation in both an anchorage-dependent and -independent manner in vitro. In addition to the decreased cell proliferation, the proportion of apoptotic cells in response to siRNA-SNORA80E transfection was increased as well as the cleavage of caspase-3 and poly [ADP-ribose] polymerase 1 (PARP1), suggesting that SNORA80E promotes cell proliferation by inhibiting apoptosis. In vivo, SNORA80E knockdown inhibits tumorigenicity. Nude mice transplanted with H1944 NSCLC cells inoculated with siRNA-SNORA80E do not develop tumors, neither ectopically nor orthotopically after a latency of 14 or 28 days, respectively, in contrast to mice engrafted with cells transfected with the control siRNA. These data support a role for SNORA80E in promoting cell proliferation, both in vitro and in vivo, and in tumorigenesis likely by inhibiting apoptosis.

**Figure 2:**
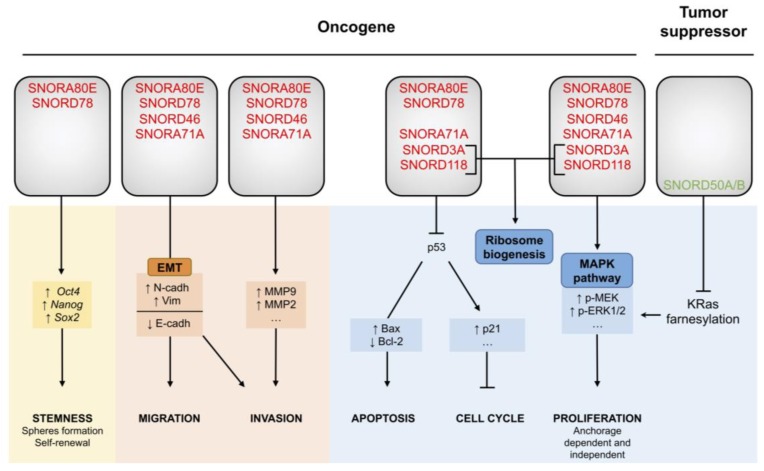
Biological functions of snoRNAs in lung cancer. snoRNAs act either as oncogenes or tumor suppressors. Experimental dysregulation of these snoRNAs affects different cellular processes (bottom panel), such as cell mobility, stemness and cell viability, by modulating different signaling pathways (middle panel). Altogether, this diagram summarizing the snoRNAs-related cellular functions reported in lung cancer, supports their key role in lung tumorigenesis and progression. snoRNAs overexpressed in lung cancer tissues compared to non-cancerous tissues are highlighted in red, underexpressed in green.

Mei and colleagues reported that the SNORA80E knockdown is associated with an accumulation of the cellular tumor antigen p53 protein in a set of NSCLC cell lines (H460, H1944 and H292). Accordingly, SNORA80E overexpression is associated with decreased p53 protein levels. Moreover, increased expression of p53 in mice xenografts was negatively correlated with SNORA80E levels, as reported by immunohistochemistry. Interestingly, apoptosis in p53-null (H299) or mutant p53 (A549) NSCLC cell lines was not affected by SNORA80E ectopic overexpression, suggesting that SNORA80E promotes cell proliferation through inhibition of apoptosis in a p53-dependent manner (Figure 2).

Furthermore, it has been reported that SNORA80E is overexpressed by 2.5-fold in CD133^+^ TICs compared to CD133- differentiated cells [59]. Downregulation of SNORA80E in H1944- and Calu-1-derived CD133^+^ cells reduces by 50% the expression of cancer stem cell-associated genes, namely POU domain class 5 transcription factor 1 (*POU5F1* or *Oct4*), nanog homeobox (*Nanog*), SRY-box transcription factor 2 (*Sox2*), neurogenic locus notch homolog 1 (*otch1*), smoothened frizzled class receptor (*Smo*) and ATP binding cassette subfamily G member 2 (*ABCG2*). Consistently, TICs transfected with the siRNA targeting SNORA80E have a reduced self-renewal capacity as observed by sphere formation and proliferation as observed by soft agar assay. After 72 h in culture, proliferation is abolished, highlighting the need for SNORA80E for cell proliferation. Therefore, SNORA80E overexpression contributes to TIC establishment and maintenance in NSCLC, certainly by regulating apoptosis. Indeed, the concomitant increase in apoptotic cells detected by cleaved caspase-3 was observed in TIC populations treated with SNORA80E-siRNA. Such findings were confirmed in vivo. Mice transfected with CD133^+^ NSCLC cells harboring the siRNA-SNORA80E are unable to develop tumors compared to control mice, underlining the pivotal function of SNORA80E in cancer stem cell properties, thus contributing to NSCLC malignancies [59].

These data demonstrate that SNORA80E acts as an oncogene. Not only is SNORA80E overexpressed in lung cancer but it also promotes cell proliferation, at least in part by inhibiting p53-induced apoptosis, and TIC formation, thereby stimulating lung tumorigenesis and aggressiveness (Figure 2). Although SNORA80E upregulation has shed light onto its role as an oncogene in prostate [71] and colorectal cancer [69], the molecular mechanisms by which SNORA80E contributes to cancer initiation and/or progression remain unclear.

### 5.2. SNORD78 Triggers Cancer Cell Plasticity

A study from Chen’s lab investigated the role of SNORD78 in lung tumorigenesis [72]. Indeed, following snoRNA profiling, SNORD78 (or U78) was considered to be an interesting snoRNA in lung cancer, as it is significantly overexpressed both in lung cancer cells and lung TICs (Table 1, Figure 1) [48,59]. Chen and colleagues first reported that SNORD78 expression level is an independent marker of poor prognosis in lung cancer. In 56 NSCLC patients, the ones harboring lung tumors expressing high levels of SNORD78 displayed a poorer overall survival than patients with lung tumors expressing low SNORD78 levels [72]. This observation supports a role for SNORD78 in lung tumorigenesis and/or progression.

Second, they showed that SNORD78 knockdown induced by the shRNA lentivirus approach in the H1975 NSCLC cell line, reduces proliferation. It was associated with an increased proportion of cells in G0/G1 and an increased protein level of the cyclin-dependent kinase inhibitors 1 (p21) and 2A (p16), two markers of G0/G1 arrest. In addition to inducing cell cycle arrest, reduction of SNORD78 expression enhanced a proportion of apoptotic cells, pro/anti-apoptotic Bax/Bcl-2 ratio and caspase-3 cleavage. Thus, SNORD78 knockdown reduces cell proliferation by inducing cell cycle arrest and apoptosis. As expected, stable overexpression of SNORD78 in A549 NSCLC cells stimulates cell division. In vivo, nude mice engrafted with H1975 NSCLC cells expressing reduced levels of SNORD78 developed tumors although smaller than the ones developed by mice engrafted with scrambled shRNA infected cells. These data support the involvement of SNORD78 in lung tumorigenesis and aggressiveness.

Transwell assays showed that SNORD78 also plays a role in the induction of NSCLC invasion. Cell motility could be acquired along an epithelial to mesenchymal transition (EMT), characterized by an enrichment in CSCs [73]. Chen’s study reported that the reduction of SNORD78 expression in the H1975 NSCLC line leads to increased expression of the epithelial marker cadherin-1 (E-cadherin) paralleled with a reduced expression of the mesenchymal markers Vimentin and cadherin-2 (N-cadherin). Moreover, they showed that SNORD78 was overexpressed especially in CD133^+^ CSC-like cells isolated from the A549 cancer cell line [72], as it has been suggested in snoRNA signatures (Table 1**) [59]. In vitro, stable silencing of SNORD78 via shRNA, resulted in fewer and smaller spheres, and in a decreased expression of a set of key stem transcription factors, including *Sox2*, *POU5F1*, *Nanog* and *Kruppel like factor 4* (*Klf4*). Interestingly, in patients, SNORD78 in combination with another biomarker, specifically predicted the appearance of lymph node metastasis. Thus, SNORD78 promotes EMT and maintenance of the stemness state of lung cancer cells to favor invasion and metastasis (Figure 2). SNORD78 might contribute to lung cancer progression through the formation of metastases that may sustain the poor survival of lung cancer patients with tumors expressing high levels of SNORD78.

Like SNORA80E, SNORD78 appears to act as an oncogene, which promotes cell proliferation by limiting apoptosis and CSC maintenance. However, the exact molecular mechanisms triggered by SNORD78 to regulate these biological functions remain to be explored. Interestingly, the individual role of SNORD78 in lung cancer is supported by the opposite effect of its host Growth Arrest Specific 5 (*GAS5*) gene. Indeed, like most snoRNAs, SNORD78 is located in an intronic region of the *GAS5* gene. It has been shown that the lncRNA GAS5 plays a tumor suppressor activity in lung cancer by sponging miRNAs with oncogenic properties [74]. Remarkably, lung cancers treated with GAS5-siRNA showed an expected reduction in GAS5 expression, independent of SNORD78 levels that remain unchanged. The dual effects of SNORD78 and lncRNA GAS5 coded by the same gene reinforce the role of SNORD78 on its own in lung tumorigenesis and progression.

### 5.3. SNORA71A: A Regulator of MAPK Signaling

To improve our understanding of the relationship between snoRNA overexpression and lung cancer, Tang et al. [67] focused on SNORA71A (U71a) located in an intron of the lncRNA SNHG17, and screened for cellular consequences of its dysregulation in NSCLC.

The relatively high level of endogenous expression of SNORA71A in A549 and PC9 lung cancer cell lines favored their use as cellular models for such investigations in vitro. The knockdown of SNORA71A in these two cell lines using an siRNA strategy induces a G0/G1 arrest, as shown by flow cytometric analyses and increases the expression of the inhibitor of G0/G1 checkpoint p27, the cyclin-dependent kinase inhibitor 1B. Moreover, SNORA71A inhibition is associated with a loss in Vimentin and a gain in E-cadherin expression, suggesting induction of EMT in both cell lines (Figure 2). In parallel, scratch wound healing assays revealed alterations of migratory and invasive properties of A549 and PC9 cells transfected with siRNA-SNORA71A. These observations support the importance of SNORA71A in the maintenance of tumorigenic characteristics of lung tumors, including cell cycle progression, EMT and migration/invasion, like SNORA80E and SNORD78.

Remarkably, in vivo, xenografted mice derived from stable A549 cells with SNORA71A knockdown display a drastic reduction in tumor growth from 350 to 50 mm^3^ compared to mice xenografted with normal A549 cells, that corresponds to an 85% reduction in tumor volume. Lung cancer tumorigenicity seems to be greatly compromised or even impossible in the absence of SNORA71A. Besides, using an available dataset from a cohort of 91 lung tumor samples [75], high levels of SNORA71A were associated with poor overall survival of lung cancer patients [67]. These data argue in favor of a role for SNORA71A in lung cancer.

Interestingly, the authors identified a disruption in the MAPK pathway in response to SNORA71A knockdown in A549 and PC9 cells. They showed a reduction in the phosphorylation of the dual specific mitogen-activated protein kinase kinase 1 (MEK) and the mitogen-activated protein kinase 1 and 2 (ERK1/2), three kinases of the oncogenic proliferative MAPK-ERK pathway (Figure 2). It has to be noted that no rescue experiment was performed to demonstrate the involvement of MAPK pathway in the cellular processes affected by SNORA71A depletion, and that the molecular mechanisms from nucleolar snoRNA dysregulation to cytoplasmic MAPK pathway extinction have not been investigated. Nevertheless, the involvement of SNORA71A in the regulation of MAPK signaling pathway offer novel opportunities to understand lung cancer biology, especially since the MAPK pathway is genetically altered in only 1% of NSCLC but is a well-known oncogenic signaling pathway in many types of cancers.

### 5.4. SNORD50A/B, Inhibitors of the Normal KRas Pathway

Most studies dedicated to investigating the contribution of snoRNAs to lung cancer were designed to identify snoRNAs the expression of which was stimulated in human lung tumors versus healthy lung samples. Such a historical approach is based on the primary objective of these pioneering studies to identify snoRNAs suitable to be used as biomarkers, a clinical test being more sensitive if there is an increased rather than a decreased expression. However, such approaches preclude the discovery of snoRNAs displaying tumor suppressive activities. Siprashvili et al. [68] took an opposite stance by conducting a large-scale study to investigate whether snoRNA genes exhibiting frequent deletion could contribute to lung cancer tumorigenesis and/or progression.

Using TCGA database, 5,473 pairs of tumoral/normal samples encompassing 21 types of cancers, which include two subtypes of lung cancer (297 LUAD and 278 LUSC), were analyzed to identify copy number alterations (CNAs) in genomic regions encoding snoRNAs. In silico analysis revealed that the somatic loss of SNORD50A (U50) and SNORD50B (U50B) genes is a common feature in different types of cancers (12 out of 21), indicating their involvement in oncogenesis. In lung cancer, SNORD50A/B are lost in 10% of lung squamous cell carcinoma and up to 30% in lung adenocarcinoma, LUAC being one of the cancers exhibiting the highest SNORD50A/B deletion frequency. These Pan-Cancer data suggest a strong correlation between SNORD50A/B and lung tumorigenesis. In agreement with observations in patients, nude mice xenografted with either A459 or NSCLC NCI-H23 lung cancer cell lines deleted in SNORD50A/B using the CRISPR method, developed larger tumors than mice injected with non-depleted cells.

As snoRNAs are known to interact with proteins, in particular within snoRNP complexes, Siprashvili et al. identified molecular interactions between SNORD50A/B and cancer-related proteins to elucidate the molecular mechanisms by which SNORD50A/B deletion promotes lung tumorigenesis. Using a small RNA-protein interaction microarray (SR-PMA) containing 9,125 recombinant proteins, high-throughput screening of proteins interacting with SNORD50A and/or SNORD50B was performed. In addition to the known molecular functions of snoRNAs in rRNA processing, SNORD50A/B are also able to interact directly with proteins that are not involved in nucleolar snoRNP complexes, such as with the GTPase KRas protein. A cross-linking and immuno-precipitation (CLIP) assay validated the interaction between recombinant KRas and SNORD50A/B and revealed an interaction between SNORD50A/B and other Ras isoforms, although to a lesser extent. Further electromobility assays also validated this unexpected binding. Diffuse residues in KRas protein and a specific 7-nt long region of the box C of the two snoRNAs contribute to the stable SNORD50A/B: KRas interaction that occurs in the cytoplasm.

In a set of four cell lines (primary keratinocytes, CHL-1 melanoma cells, Cal27 head and neck squamous cell carcinoma cells and ME180 cervical carcinoma) harboring a functional Ras-Raf-MAPK pathway, reduction of SNORD50A/B expression by specific Antisense Oligonucleotides (ASOs) triggers ERK1/2 and MAPK activation and reduces cell proliferation in vitro. Furthermore, total abolition of SNORD50A/B expression using the CRISPR technology in CHL-1 melanoma (wild-type KRas) and two A549 and NCI-H23 lung cancer cell lines (mutant KRas) also resulted in an increase in ERK1/2 phosphorylation and in cell proliferation. SNORD50A/B overexpression in both wild-type and mutant KRas cells led to a decrease in ERK1/2 phosphorylation and limited cell growth and proliferation. Hence, SNORD50A/B acts as a negative regulator of the KRas pathway irrespective of its mutational status, and affects tumorigenic capacities of cancer cells, including lung cancer cells (Figure 2). Interestingly, the specific interaction between SNORD50A/B and KRas induces the inhibition of interaction between KRas and the farnesyltransferase (FTase), which usually leads to the activation of the KRas protein through farnesylation and prenylation. The current model suggests that when SNORD50A/B is depleted, FTase then binds to KRas, leading to its activation and downstream signaling pathway that could provide support for the ERK1/2 phosphorylation observed in cell lines lacking SNORD50A/B. Thus, SNORD50A/B act as tumor suppressors probably by preventing KRas activation.

Based on these data, it appears that the depletion of SNORD50A/B often found in patient tumor samples is a key step for lung tumor initiation by activating the oncogenic KRas pathway, the genomic alterations of which represent about 25% of lung adenocarcinomas [42]. SNORD50A/B is the only example so far of a snoRNA with suppressive activity in lung cancer. Moreover, those data illustrate the possibility for snoRNAs to interact directly with cytoplasmic proteins in addition to nucleolar proteins involved in snoRNPs. The physical binding of a snoRNA with an unexpected protein has already been observed. SNORD46, a C/D box snoRNA, is overexpressed in many cancers [3]. Its inhibition using antisense locked nucleic acid (LNA) Gapmer technology in the NSCLC A549 cell line resulted in decreased cell viability, migration and invasion, suggesting a function for SNORD46 it these cellular mechanisms. In addition, like SNORD50A/B, SNORD46 interacts with oncoproteins. Experimental identification of proteins interacting with a synthetized SNORD46 through RNA-pull down and mass spectrometry analysis (MS), demonstrated an interaction with the pappalysin-1 (PAPP1) protein, a secreted metalloprotease, probably unveiling unknown functions of snoRNAs in non-canonical localization.

### 5.5. SNORD3A and SNORD118: From Ribosome Biogenesis to Tumorigenesis

Since a majority of the studies investigating the contribution of snoRNA dysregulation in lung tumorigenesis did not decipher the direct snoRNA-related molecular mechanism but rather provided an associative evidence between snoRNA dysregulation and cancer cell behavior, Langhendries et al. [19] from D. Lafontaine’s lab attempted to address this concern by focusing on the primary role of snoRNAs in pre-rRNA processing during tumorigenesis.

SNORD3A (U3) and SNORD118 (U8) belong to the small fraction of C/D box snoRNAs participating in the cleavage of the 47S pre-rRNA into the three main rRNA species (5.8S, 18S and 28S) prior to their assembly into ribosomal proteins in eukaryotic species. Dysregulation in the expression levels of these two snoRNAs has never been steadily detected in lung cancer. However, since inhibition of SNORD3A and SNORD118 impedes mammary tumorigenesis [27], Langhendries et al. investigated their individual role in tumorigenesis using two cellular models, namely human lung (H1944) and breast (MCF-7) cancers. Only data related to lung cancer are presented herein.

In response to reduced SNORD3A or SNORD118 levels, p53 and p21 proteins accumulate (up to 25-fold after 24 h) as evidenced by Western blot, a regulation dependent on the presence of the ribosomal proteins RPL5 and RPL11. It is associated first with a G1 blockade, around 80% of NSCLC H1944 cells being arrested only after 24 h of ASO treatment, and second with an increased proportion of apoptotic cells associated with progressive dislocation of the nucleolus. Thus, reduction of SNORD3A or SNORD118 levels promotes a p53 suppressive response in a ribosomal protein-dependent manner. Such response has been extensively studied and corresponds to a classical response termed ribosomal stress (or nucleolar stress), where defects in ribosome biogenesis, including defects in pre-rRNA cleavage, induce cell cycle arrest and apoptosis in a p53-dependent manner through disruption of the interaction between p53 and the human double minute 2 (Hdm2) protein by ribosomal proteins (for review see [76,77]).

Furthermore, SNORD3A and SNORD118-depleted H1944 lung cancer cells lose their ability to form colonies in vitro using soft agar assay. While reduction of SNORD3A levels diminished colony formation by 75%, the effect of SNORD118 knockdown is even stronger since it completely prevents colony growth. Injection of cells previously treated with SNORD3A or SNORD118 ASO inhibitor in nude mice corroborate these results. Among the 12 mice engrafted with SNORD118-depleted H1944 cells, no tumor was visible using either necked eye or PET (positron emission tomography) two months after engraftment, although the scramble treated H1944 cells led to the formation of visible and palpable tumors. The other set of 12 mice transplanted with H1944 lung cancer cells expressing reduced SNORD3A levels in response to ASO treatment, developed tumors 24 days after transplant though these were 500-fold smaller than control tumors.

Polysome analysis of H1944 cells treated with specific ASO targeting either SNORD3A or SNORD118, revealed a reduction in 40S (i.e., small ribosome subunit) and 60S (i.e., large ribosome subunit) peaks, respectively. As a consequence, a reduction in polysomes (i.e., mRNAs bound by ribosomes) is observed, indicative of a reduction in ribosome production however in a different extent. Indeed, some polysomes are still visible in response to SNORD3A knockdown, while they are largely absent in response to SNORD118 depletion. Such a difference may be due to the fact that knockdown expression of these snoRNAs alters different steps of the pre-rRNA cleavage as shown by Northern blot assays (SNORD3A: 5′-ETS and ITS1 leading to 18 rRNA; SNORD118: ITS2 and 3′-ETS involved in 28S rRNA), thus driving the accumulation of inappropriate rRNA intermediates. Hence, SNORD3A and SNORD118 do have key functions in maintaining human ribosome biogenesis in cancer cells.

Altogether, it appears that SNORD118 is more tumorigenic than SNORD3A. The difference observed in their individual impact on ribosome biogenesis might explained such a difference in tumorigenic activity, the 60S defects associated with SNORD118 depletion may have more impact on tumorigenesis than the 40S defects associated with SNORD3A although it remains to determine by which mechanism. Nevertheless, both SNORD3A and SNORD118 directly contribute to lung tumorigenesis by promoting ribosome production necessary to maintain cell growth and the high proliferative rate of cancer cells.

## 6. The Future of snoRNAs in the Field of Lung Cancer

The involvement of snoRNAs in lung cancer tumorigenesis is paving the way for novel studies investigating the pathophysiology of lung cancer. Indeed, it appears that studying genomic alteration is no longer sufficient to understand both lung tumorigenesis and progression. Moreover, it emerges. It only starts to emerge that alteration in gene expression might mediate resistance to targeted therapies [41]. In parallel, the demonstration that snoRNA expression varies in lung cancer tissues challenges the former dogma, according to which snoRNAs were considered to be effectors of housekeeping functions in ribosome biogenesis [6]. SnoRNAs are mostly overexpressed in lung cancer tissues compared to non-cancerous samples, while only a few of them are downregulated in lung tumors (Table 1). Mechanistic examination of such dysregulation in lung cancer cells argues in favor of oncogenic activities for most snoRNAs.

Overexpressed snoRNAs act as oncogenes activating different survival pathways such as MAPK or KRas, likely through inhibition of p53-mediated apoptosis. Experimental inhibition of some individual snoRNAs abolished lung tumorigenesis in vitro and in vivo. Knowing the emerging role of cancer stem cells in lung tumorigenesis and progression, both by promoting metastases and resistance to targeted therapies, the importance of snoRNAs in promoting and maintaining self-renewal properties in lung cancer illustrates the major role of snoRNAs in lung cancer malignancies. To date, a single study accumulates evidence regarding the tumor suppressive role of snoRNAs, attributed to *SNORD50A* and *SNORD50B*, which are deleted in lung tumors [68]. However, it is expected that many more snoRNAs may have tumor suppressive effects.

Research in this field is unquestionably at its infancy and requires further insight. Preliminary data relied on promising findings that need to be completed, in particular regarding the molecular mechanisms by which snoRNAs contribute to lung cancer tumorigenesis and progression. The notion that expression of snoRNAs can be altered in cancer, thus questioning their housekeeping functions, has to be paralleled with the emerging field of epitranscriptomics [15,78,79]. Indeed, the canonical role of snoRNAs in catalyzing rRNA chemical modifications (i.e., 2′-*O*-ribose methylation and pseudo-uridylation) has not yet been explored, although it appears that rRNA chemical modifications play a key role in regulating translation [15]. Indeed, alterations of rRNA 2′-*O*-ribose methylation directly affect translation of some mRNAs, which contain particular cis-regulator elements in their 5′ UTR (i.e., Internal Ribosome Entry Sites or IRES), such as the Insulin-like Growth Factor 1 Receptor (*IGF1R*) or Myc proto-oncogene (*MYC*), in breast cancer and leukemia [80,81,82]. However, the relationship between ribosome biogenesis and lung cancer is supported not only by the role of the SNORD3A- and SNORD118-induced 47S pre-rRNA cleavage in lung cancer proliferation, but also by the recent demonstration that rRNA synthesis is required to drive *KRAS*-*TP53* lung tumorigenesis [83]. One could speculate that malignancy mediated by snoRNAs in lung cancer is driven by the fine-tuning of ribosomes. However, it appears that snoRNAs might exert yet undescribed non-canonical functions outside of the nucleus, since some studies demonstrated snoRNA binding to extra-nuclear proteins and reported the role of some “orphan” snoRNAs in mRNA metabolism [3,68].

Studying snoRNAs in lung cancer may pave the way for innovative clinical applications, both as biomarkers and therapeutic targets. snoRNAs have singular intrinsic biochemical features, ideal as a source of non-invasive lung cancer biomarkers. Indeed, snoRNAs are released through exosomal vesicles or after cell death in circulating fluids such as sputum [60] or plasma [48], where they are stably and readily detected in a non-invasive manner. Precise identification of snoRNA signatures during the course of the disease may provide major indications of the subtype and the stage of the disease, but also predict relapse [3]. Discovering novel signatures will rely on RNA-seq approaches, although nomenclature issues inherent to both diverse snoRNA annotations and snoRNA identity based on a unique 10–20 nt sequence, have still to be addressed. From the therapeutic point of view, it could be hypothesized that snoRNAs can be efficient targets to reduce lung tumor growth, as it has been observed in in vitro and in vivo experiments. The transfer of experimental findings on the inhibition of snoRNAs to novel clinical therapeutic approaches may be highly promising. RNA therapies began in the 1990s, the first US Food and Drug Administration (FDA) approved RNA-based therapy using antisense oligonucleotide to cure cytomegalovirus retinitis having been released in 1998 (Nature outlook, RNA therapies 2019). MicroRNA themselves can be used as efficient drugs as demonstrated by several clinical trials, including in lung cancer (NCT02369198) [84]. Moreover, tools could be designed to repress snoRNAs in a specific manner, including antisense oligonucleotides or miRNAs [84,85].

## 7. Conclusions

The literature dedicated to snoRNAs in lung cancer, although limited today, highlights the biological and clinical outcomes that may arise from studying snoRNAs. In particular, confronted with the limitations presented by current therapies used in lung cancer management and the associated elevated morbidity in lung cancer patient populations, snoRNAs may become pivotal elements to improve our understanding of lung cancer and relevant multimodal tools to improve cancer patient management from their diagnosis to their treatment.

## Figures and Tables

**Table 1:** snoRNA signatures identified in lung cancer patients.

References	Sample Type	Cohort Characteristics	Cancer Sub-Type (*N*)	Stage (*N*)	Total snoRNAs Analyzed	Technology	Fold Change Threshold	*p*-Value Threshold	Number of snoRNAs in Signatures	snoRNA Signature
**a. snoRNA signatures in normal vs tumors samples**										
**Liao et al. 2010** [48] **F. Jiang lab**	Lung Tissues	22 NSCLC patient samples/22 matched normal patient samples	LUSC (11) LUAD (11)	Stage I (22)	352	GeneChipR Arrays (Affymetrix)	≥|1.0-fold change|	*p* < 0.01	31	SNORA18, SNORA21, SNORA80E, SNORA73B, SNORD1C, RNU3P2, SNORD13, SNORD25, SNORD29, SNORD31, SNORD33, SNORD34, SNORD35A, SNORD36C, SNORD38B ^(1)^, SNORD44, SNORD49A, SNORD55, SNORD66, SNORD76, SNORD78, SNORD83B, SNORD88A, SNORD88C, SNORD95, SNORD96A, SNORD100, SNORD104, SNORD110, SNORD116-26
							≥|1.5-fold change|	*p* < 0.01	6 *	SNORA80E, SNORA73B, SNORD33, SNORD66, SNORD76, SNORD78
	Plasma	37 NSCLC patient samples/37 non-cancerous samples (22 healthy donors and 26 COPD)	LUSC (16) LUAD (21)	Stage I (10) Stage II (12) Stage III-IV (15)	6 *	RT-qPCR	NA	*p* < 0.01	3	SNORD33, SNORD66, SNORD76
**Gao et al. 2015** [49] **F. Jiang lab**	Lung Tissues	12 NSCLC patient samples/12 matched normal patient samples	LUSC (6) LUAD (6)	Stage I (12)	458	Deep sequencing (Illumina^®^ Genome Analyzer IIx)	≥|2.0-fold change|	*p* ≤ 0.001	68	SNORA12, SNORA14A, SNORA18, SNORA21, SNORA34, SNORA38B, SNORA39, SNORA80E, SNORA47, SNORA57, SNORA64, SNORA66, SNORA68, SNORA70 ^(2)^, SNORA71A, SNORA71C, SNORA73B, SNORA75, SNORA78, SNORA80, SNORA80B, SNORD3A, SNORD3B-1, SNORD3B-2, SNORD3D, SNORD10, SNORD28, SNORD33, SNORD66, SNORD74 ^(1)^, SNORD76, SNORD78, SNORD80, SNORD96A, SNORD113-5, SNORD113-7, SNORD113-8, SNORD114-11, SNORD114-13, SNORD114-20, SNORD114-25, SNORD114-26, SNORD114-28, SNORD115-2, SNORD115-9, SNORD115-10, SNORD115-12, SNORD115-15, SNORD115-17, SNORD115-18, SNORD115-19, SNORD115-23, SNORD115-32, SNORD115-34, SNORD115-38, SNORD115-40, SNORD115-41, SNORD115-42, SNORD116-18, SCARNA1, SCARNA12, SCARNA20 ^(1)^, SCARNA21, SCARNA23
							≥|3.0-fold change|	*p* ≤ 0.001	29	SNORA12, SNORA14A, SNORA21, SNORA34, SNORA38B, SNORA39, SNORA47, SNORA64, SNORA66, SNORA68, SNORA70, SNORA71A, SNORA71C, SNORA75, SNORA78, SNORA80, SNORA80B, SNORD10, SNORD28, SNORD66, SNORD74, SNORD80, SNORD96A, SNORD113-7, SNORD113-8, SNORD114-20, SNORD114-28, SNORD115-32, SNORD115-41
**Su et al. 2015** [60] **F. Jiang lab**	Sputum	59 NSCLC patient samples/ 61 cancer-free smoker samples	LUSC (28) LUAD (31)	Stage I (29) Stage II (30)	6 *	RT-qPCR	NA	*p* < 0.05	4	SNORA80E, SNORD33, SNORD66, SNORD78
**Gong et al. 2017** [3] **L. Han lab**	Lung Tissues	91 NSCLC patient samples/91 matched normal patient samples	LUSC (45) LUAD (46)	NA	465	miRNA-seq (TCGA data set)	≥|2.0-fold change|	*p* < 0.05	46	SNORA21, SNORA56, SNORA71B, SNORA71D, SNORA73A, SNORA73B, SNORA84 ^(3)^, SNORD1A, SNORD9, SNORD10, SNORD11, SNORD12B, SNORD12C, snoU13, SNORD13, SNORD14C, SNORD15A, SNORD15B, SNORD16 ^(3)^, SNORD18, SNORD18C, SNORD19B, SNORD30, SNORD34, SNORD35A, SNORD35B, SNORD36C, SNORD41, SNORD44, SNORD45C, SNORD46, SNORD58C, SNORD72, SNORD75, SNORD76, SNORD77, SNORD78, SNORD80, SNORD82, SNORD83A, SNORD83B, SNORD88A, SNORD96A, SNORD102, SNORD114-14 ^(4)^, SCARNA5
**b. CSC-snoRNA signatures in patients**										
**Mannoor et al. 2014** [59] **F. Jiang lab**	Lung Tissues	ALDH^+^ cells (TICs) sorted from 22 NSCLC tumor tissues/ALDH^−^ cells sorted from matched 22 NSCLC tumor tissues	NA	NA	352	GeneChipR Arrays (Affymetrix)	≥|3.0-fold change|	*p* < 0.01	22	SNORA3, SNORA18, SNORA80E, SNORA61, SNORA62, SNORD1C, SNORD14E, SNORD33, SNORD34, SNORD36C, SNORD38B, SNORD44, SNORD55, SNORD66, SNORD73B, SNORD76, SNORD78, SNORD83B, SNORD88A, SNORD96A, SNORD110, SNORD116-26
**c. Specific snoRNAs in lung compare to other cancer types**										
**Pan et al. 2019** [51] **Y-D. Cai lab**	Lung Tissues		LUSC (521) LUAD (559)	NA	459	miRNA-seq (TCGA data set and Gong et al. 2019 [3] data)	NA		10 ^(5)^	SNORA31 (ACA31) ^(3)^, SNORA47 (HBI-115) ^(3)^, SNORD7 (mgU6-47) ^(4)^, SNORD81 (U81) ^(4)^, SNORD83B (U83B) ^(3)^, SNORD99 (HBII-420) ^(4)^, SNORD115 ^(4)^, SNORD115-32 (HBII-52-32) ^(4)^, SNORD123 ^(3)(4)^

* Correspond to the 6 top snoRNAs composing the signature defined in lung tissues by Liao et al., 2010; ^(1)^ found twice in the data however with different fold changes; ^(2)^ found three times in the data however with different fold changes; ^(3)^ specific to LUSC in this signature; ^(4)^ specific to LUAD in this signature; ^(5)^ complete signature not available; red: overexpressed in lung cancer tissues; green: underexpressed in lung cancer tissues.

## Supplementary Materials

The following are available online at https://www.mdpi.com/2073-4409/9/3/541/s1, Table S1: snoRNA nomenclature.
